# Production of Ceramics/Metal Oxide Nanofibers via Electrospinning: New Insights into the Photocatalytic and Bactericidal Mechanisms

**DOI:** 10.3390/ma16145148

**Published:** 2023-07-21

**Authors:** Jari S. Algethami, Touseef Amna, Laila S. Alqarni, Aisha A. Alshahrani, Mohsen A. M. Alhamami, Amal F. Seliem, Badria H. A. Al-Dhuwayin, M. Shamshi Hassan

**Affiliations:** 1 Department of Chemistry, College of Science and Arts, Najran University, Najran 11001, Saudi Arabia; 2 Promising Centre for Sensors and Electronic Devices (PCSED), Advanced Materials and Nano-Research Centre, Najran University, Najran 11001, Saudi Arabia; 3 Department of Biology, College of Science, Al-Baha University, Albaha 65799, Saudi Arabia; 4 Department of Chemistry, College of Science, Imam Mohammad Ibn Saud Islamic University (IMSIU), Riyadh 11432, Saudi Arabia; 5 Department of Chemistry, College of Science, Al-Baha University, Albaha 65799, Saudi Arabia

**Keywords:** bactericidal mechanism, Zr_0.5_Sn_0.5_TiO_3_/SnO_2_ composite, ceramics nanofibers, environmental pollution, photocatalysis

## Abstract

Environmental pollution is steadily rising and is having a negative influence on all living things, especially human beings. The advancement of nanoscience in recent decades has provided potential to address this issue. Functional metal oxide nanoparticles/nanofibers have been having a pull-on effect in the biological and environmental domains of nanobiotechnology. Current work, for the first time, is focusing on the electrospinning production of Zr_0.5_Sn_0.5_TiO_3_/SnO_2_ ceramic nanofibers that may be utilized to battle lethal infections swiftly and inexpensively. By using characterizations like XRD, FT–IR, FESEM, TEM, PL, and UV–Vis–DRS, the composition, structure, morphology, and optical absorption of samples were determined. The minimum inhibitory concentration (MIC) approach was used to investigate the antibacterial activity. Notably, this research indicated that nanofibers exert antibacterial action against both Gram-positive and Gram-negative bacteria with a MIC of 25 µg/mL. Furthermore, negatively charged *E. coli* was drawn to positively charged metal ions of Zr_0.5_Sn_0.5_TiO_3_/SnO_2_, which showed a robust inhibitory effect against *E. coli*. It was interesting to discover that, compared to pure TiO_2_, Zr_0.5_Sn_0.5_TiO_3_/SnO_2_ nanofibers revealed increased photocatalytic activity and exceptional cyclability to the photodegradation of Rhodamine B. The composite completely degrades dye in 30 min with 100% efficacy and excellent (97%) reusability. The synergetic effects of Zr_0.5_Sn_0.5_TiO_3_ and SnO_2_ may be responsible for increased photocatalytic and bactericidal activity.

## 1. Introduction

Environmental pollution is not a recent phenomenon, but it continues to be the greatest threat to mankind and a major factor of various illnesses and mortality. Worldwide urbanization, industrialization, mining, and excavation are human activities that have the greatest impact on the environment. Indisputably, water pollution is also a big concern in today’s world and a serious hazard that causes several problems for humans and the environment. Water pollution must be halted since it has been harming land and water species for years by entering lakes, rivers, streams, and seas. There are reports in the literature that an increasing number of individuals lack access to fresh, hygienic water. The water industry currently uses certain conventional chemical disinfectants, but it is alarming that many of these are carcinogens. Additionally, the need for more effective substitutes is growing as microorganisms’ resistance to conventional chemical disinfectants increases. *E. coli and Enterococci* are found in high numbers in the feces of animals; they are regarded as reliable indicators of potential damage to human health from fecal pathogens in fresh, untreated sewage [1]. Furthermore, swimming and boating are prevalent pleasurable outdoor water leisure activities that are deeply ingrained in western society [2]. However, these actions produce a significant financial expense owing to the risk of associated waterborne infections produced by harmful microbes [2]. Pathogens that cause waterborne diseases are commonly found in recreational waters due to fecal contamination [3]. The removal of pathogenic bacteria from water is a critical operation for drinking and sanitation systems, particularly in light of the increased occurrence of water-borne diseases [4]. Water pollution control has become increasingly important in recent years. Considering the growing ecological pollution and energy crises, photocatalysis, a green technique, exhibits a vital role in the solar energy transformation and the disintegration of organic pollutants. The advancement of applied microbiology and nanotechnology in recent decades has provided potential to address this issue [5]. Nanotechnology is a flourishing scientific discipline with numerous applications, including medicine, semiconductor manufacture, environmental remediation, and the production of personal care items. Because of its wide range of uses, there has been a tremendous increase in the manufacture of customized functional nanomaterials. The developed nanomaterials are distinguished by having one or more dimensions smaller than 100 nm [6]. Recent breakthroughs in nanotechnology have made it possible to effectively produce, characterize, and modify the functional properties of nanoparticles for biomedical purposes as well. Functional nanomaterials are having an impact on biological and therapeutic applications [7]. Nanoparticles display distinct features, since they are different in terms of size, distribution, and form [8,9]. Recent decades have seen an increase in research into the synthesis and characterization of nanoparticles owing to their numerous uses, notably in the fields of cosmetics, diagnostics, health care, environmental cleaning, pharmaceuticals, medical implants, and the energy sector [10,11]. Furthermore, in the last few decades, there has been an increase in the production of functional one-dimensional (1D) nanostructures, for instance, nanofibers, nanowires, nanotubes, nanorods, nanobelts, and so on; this is owing to their distinctive chemical and physical chattels that differ from their bulk equivalents and their potential applications in nanodevices as components and intersects [12]. In comparison to their film and particulate counterparts, nanofibrous photocatalysts not only have a large specific surface area, which allows their surface active sites to be more efficiently available to reactants, but they also have a high length-to-diameter ratio, which facilitates photocatalyst separation [13]. Electrospinning is a simple, effective, and convenient method of producing polymer/polymer-containing inorganic materials and inorganic/ceramics fibers [14]. Interestingly, it was also revealed that electrospun nanofibrous photocatalysts possess excellent photocatalytic efficiency and reuse proficiency [15].

The TiO_2_ is frequently regarded as the most ideal material for prevalent ecological uses because of its strong photoelectrochemical activity and stability against photo and chemical corrosion [12]. However, due to the inefficient usage of photogenerated electron–hole pairs, more research is required to increase the activity of this semiconductor. Suppressing the recombination of photogenerated electrons and holes in a semiconductor particulate system is critical for boosting the net charge transfer efficiency in photocatalysis and solar cells [2]. In order to achieve this goal, a variety of approaches have been applied, including the alteration of the TiO_2_ surface using noble materials [16,17] and the pairing of two semiconductor particles using unlike Fermi planes [18].

Owing to the structural similarity between the two oxides, the TiO_2_–SnO_2_ system has been the focus of several investigations, including of semiconductors [19]. The conduction band of SnO_2_ works as a drain for photogenerated electrons when two semiconductor particles are linked [18,20]. Because photogenerated holes flow in the conflicting direction, they concentrate in the TiO_2_ particle’s valence band, resulting in the proficient spatial parting of photogenerated charges and the suppression of recombination. Furthermore, the Sn dopant increases the photoinduced charge rate of TiO_2_ [21]. To enhance the thermal stability and photocatalytic action, metal oxides such as ZnO_2_, SiO_2_, La_2_O_3_, etc., have also been utilized [14,15]. Moreover, the distinctive features of zirconia, such as the high refractive index, broad optical band gap, low absorption, and dispersion in the visible and near-infrared spectrum areas [22,23] are appealing. Previously, it was observed that adding ZrO_2_ to TiO_2_ improved the phase stability, surface area and ultimately photocatalytic effects [24,25,26]. Interestingly, earlier works have also reported the formation of zirconium–stannate titanate (Zr_1−x_Sn_x_TiO_4_) thin films via the spin coating technique [27]. Additionally, the use of biomedical-grade ZrO_2_ demonstrates the potential mechanical properties of oxide ceramics. It is emerging as an important catalyst in a variety of photocatalytic and piezoelectric applications, and in ceramics production and dentistry for healing purposes [28]. Despite its potential medicinal uses, few investigations on the significance of ZrO_2_ as an antimicrobial, anticancer, and antioxidant agent have been published. In this regard, Gupta et al. demonstrated the cytotoxic effect of ZrO_2_ nanoparticles on A549 cells and their antibacterial action on *E. coli* [29]. There have been numerous cases of using doping in the ZrO_2_ structure to enhance its antibacterial action. Imran and colleagues published a study on the antibacterial properties of Fe_3_O_4_-doped ZrO_2_ nanoparticles. Their bactericidal effect against *S. aureus*, *E. coli* and *B. subtilis* has been increased by doping [30]. Jangra et al. discovered that ZrO_2_ has antibacterial action solely against *E. coli*, whereas ZrO_2_ complexes are active against both *E. coli* and *S. aureus* [31]. Another study looked at the antibacterial efficacy of ZrO_2_ nanoparticles against Staphylococcus strains. The findings showed their low activity at squat concentrations, and vice versa, both in the case of *S. aureus* and the mutant, respectively [32].

Conclusively, considering the noteworthy properties of SnO_2_, ZrO_2_ and TiO_2_ in the current investigation, we aimed to design highly efficient Zr_0.5_Sn_0.5_TiO_3_/SnO_2_ (ZSTS) nanofibrous photocatalysts via the electrospinning method. Hitherto, there has been no published report regarding the synthesis, characterization and, more to the point, the bactericidal effect of these composite ceramics nanofibers. Herein, a first attempt was made to fabricate high-surface-to-area-ratio ZSTS ceramics nanofibers. Moreover, these high-aspect-ratio nanofibers have been investigated for their photocatalytic and antibacterial activities. These novel ceramics/metal oxide nanofibers will serve as the material of choice for environmental remediation, especially when aiming to control the quality of wastewater. Our research demonstrates that multifunctional ZSTS nanofibers may be a commendable candidate for use in health care and environmental pollution control systems.

## 2. Materials and Methods

### 2.1. Materials

Sigma-Aldrich from the United States supplied the polyvinyl acetate (PVAc, *Mw* = 500,000 g/mol), tin(II)2-ethylhexanoate (98%), and zirconyl chloride octahydrate (98%). N, N-dimethylformamide (DMF, 99.5 assay) and titanium isopropoxide (TIP, 98.0 test) were bought from Japan’s Showa Chemicals Ltd. And Junsei Company. *E. coli* and *S. aureus* were bought from ATCC to test the antibacterial activity. Unless otherwise stated, all the reagents employed were of analytical quality.

### 2.2. Preparation of TiO_2_ Nanofibers

The PVAc solution (18 wt%) was made by dissolving PVAc powder in DMF using magnetic stirring for 8 h at room temperature. In a separate vial, 5 g of titanium isopropoxide was mixed with a few drops of acetic acid until the mixture was transparent. The solution was then aggressively agitated while being gently blended with 6 g of PVAc solution. The resultant solution was put into a 10 mL syringe using a stainless steel needle. The counter electrode was a ground iron drum coated with a polyethylene sheet, and the positive terminal was a copper pin inserted into the solution from a high-voltage generator. In order to keep the solution in the capillary, the angle of inclination was adjusted. This solution received a 20 kV voltage. The distance between the collector and the needle tip of the syringe was set at 18 cm. The synthesized composite ceramics fiber mat was first dried at 80 °C for 24 h under vacuum before being calcined at 600 °C for 2 h in an environment of air at a heating rate of 2 °C/min.

### 2.3. Preparation of Zr_0.5_Sn_0.5_TiO_3_/SnO_2_ Ceramics Nanofibers

Briefly, titanium isopropoxide (1 mM, 2.84 g) was continuously stirred into the PVAc solution with 1 mL of acetic acid. The solution was then supplemented with Tin(II)2-ethylhexanoate (1 m mole) and Zirconyl chloride octahydrate (ZrOCl_2_.8H_2_O, 0.5 mM) in 3 mL of ethanol. The resultant mixture was electrospun and calcined under the same circumstances as those described for making titania nanofibers.

### 2.4. Material Characterization

By using X-ray powder diffraction (XRD, Rigaku, Cu K = 1.5406 Å), the plain and ceramics/metal oxide nanofibers were identified for crystallinity. An energy-dispersive X-ray spectrometer (EDX) was used to test the chemical components of the products, while field-emission scanning electron microscopy (FESEM; JEOL JSM-7500FA, JEOL, Tokyo, Japan) and transmission electron microscopy (TEM; JEOL, 2011, JEOL, Tokyo, Japan) were used to investigate the morphologies and structures. Thermogravimetric analysis (TGA) was performed in an air atmosphere from room temperature to 800 °C with a heating rate of 10 °C/min. On a UV–Vis spectrophotometer (UV-2550, Shimadzu, Kyoto, Japan), the fibers’ UV–DRS (Ultraviolet diffuse reflectance) spectroscopy was recorded. A Nicolet Nexus 670 FTIR spectrophotometer was used to perform Fourier-transform infrared spectroscopy (FTIR) at a resolution of 4 cm^−1^. The photoluminescence (PL) spectra were obtained using a Hitachi, Japan, F-7000 fluorescence spectrophotometer with a 325 nm excitation wavelength.

### 2.5. Photocatalytic Experiment

The rate of photodegradation of the ceramics nanofibers for Rhodamine B dye in deionized water was used to measure its photocatalytic activity. Following a typical procedure, 250 mL of Rhodamine B solution (20 ppm) was mixed with 0.1 g of photocatalyst using a magnetic stirrer for 30 min in the dark to reach an equilibrium between the dye and the catalyst before being exposed to UV light (λ = 254 nm). Every 5 min, a sample was taken, and to remove any potential photocatalytic particles, it was centrifuged. The degradation rate was then computed after the concentration of Rhodamine B was obtained via its UV–vis absorption at 553.5 nm (Shimadzu UV 1700, Tokyo, Japan).

### 2.6. Computation of the Antibacterial Potential of TiO_2_ and Zr_0.5_Sn_0.5_TiO_3_/SnO_2_ (ZSTS) Nanofibers

The antibacterial activity was scrutinized using a conventional process by exposing pathogenic organisms cultivated at pH 7.4 and an rpm of 150 to different concentrations of TiO_2_ and ZSTS nanofibers. The aqueous solution containing TiO_2_ and ZSTS nanofibers was exposed to UV light for 30 min prior to being infused with bacteria, as described elsewhere [33]. Bacteria such as *S. aureus* and *E. coli* were grown in a Petri dish. To determine the minimal concentration for bacterial growth inhibition, the TiO_2_ and ZSTS nanofiber suspension was tested at several concentrations (0, 25, 50 and 100 µg/mL). Utilizing the earlier reported approach for the MIC, an initial evaluation of the antibacterial activity was conducted [34]. Briefly, 20 mL of agar with 1 mL of the aforementioned microbial culture was placed in Petri dishes. Separate doses for the two strains were administered. At 37 °C, the Petri plates were incubated for 24 h. In order to achieve a turbidity of 1 × 10^6^ CFU/mL, 5 mL of nutrient broth was injected with a loopful of overnight microbial cells from the two strains that were chosen from the agar plates. As previously defined, pure TiO_2_ and various concentrations of the ZSTS nanofibers were irradiated using UV and tested. An Ultraviolet (UV)–Visible spectrophotometer (UV-2550, Shimadzu, Japan) was deployed to measure the optical density (OD), which was recorded every three hours during an incubation period.

## 3. Results and Discussion

The one-dimensional nanometer-scale shape and the distinctive chemical and electrical characteristics of metal oxide nanofibers have attracted a great deal of scientific attention. It has been shown that they have a range of uses in light-emitting diodes, gas sensors, liquid crystal spectacles and cosmological cells. A quick and simple approach to creating nanofibers with a diameter many orders of scale smaller to those made via traditional spinning procedures is electrospinning. Electrospun nanofibers have several uses in photocatalysis, filtration, protective fabrics, battery cells, and biomedicine [35]. The potential of nanofibers to stop bacterial development or infection is also very appealing and a crucial function. In this study, we efficaciously synthesized ZSTS nanofibers via the electrospinning procedure for the first time.

**Figure 1:**
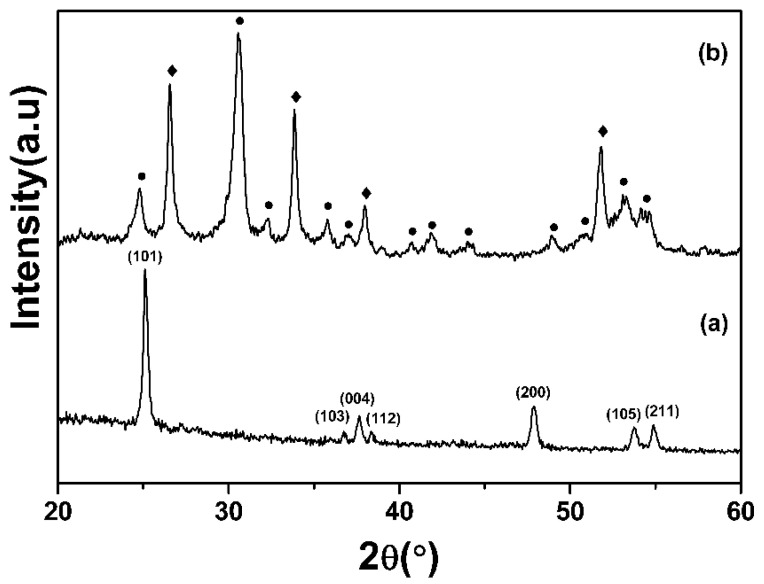
XRD spectra of (a) TiO_2_, and (b) ZSTS nanofibers (“●” represents Zr_0.5_Sn_0.5_TiO_3_, whereas “◆” represents SnO_2_).

The crystal structure report associated with TiO_2_ nanofibers and the ZSTS nanofibrous composite has been investigated via XRD, as demonstrated in Figure 1. Spectrum a in Figure 1 shows that all diffraction crests can be assigned to anatase TiO_2_ (JCPDS No. 89−4921). Meanwhile, all the diffraction peaks shown in spectrum b of Figure 1 can be well designated to cubic Zr_0.5_Sn_0.5_TiO_3_ (JCPDS No. 34−0033), and the tetragonal structure of SnO_2_ (JCPDS No. 71-0652). Each peak of SnO_2_ corresponds to the (110), (101), (200), (211) and (301) plane [36]. The sharp diffraction peaks of both Zr_0.5_Sn_0.5_TiO_3_ and SnO_2_ indicate their commendable crystallinity. No traces of other phases are examined, confirming the high purity of the samples. Thus, it is confirmed that the material formed is a fibrous ZSTS nanocomposite.

**Figure 2:**
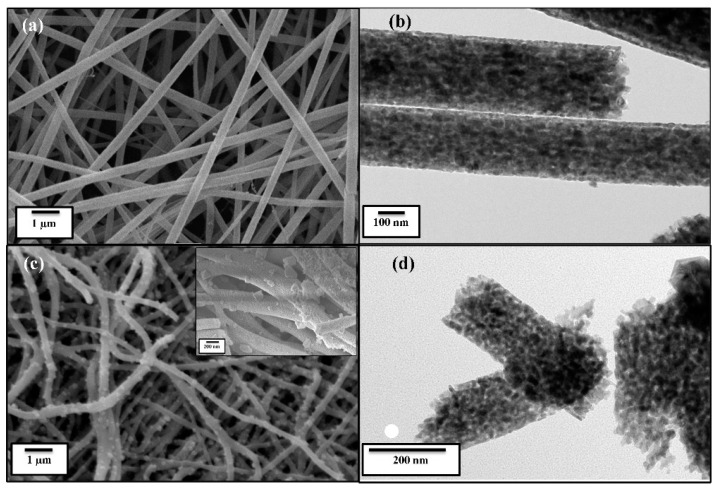
FESEM and TEM images of (**a**,**b**) TiO_2_ and (**c**,**d**) ZSTS nanofibers (inset shows FESEM of ZSTS at high magnification).

**Figure 3:**
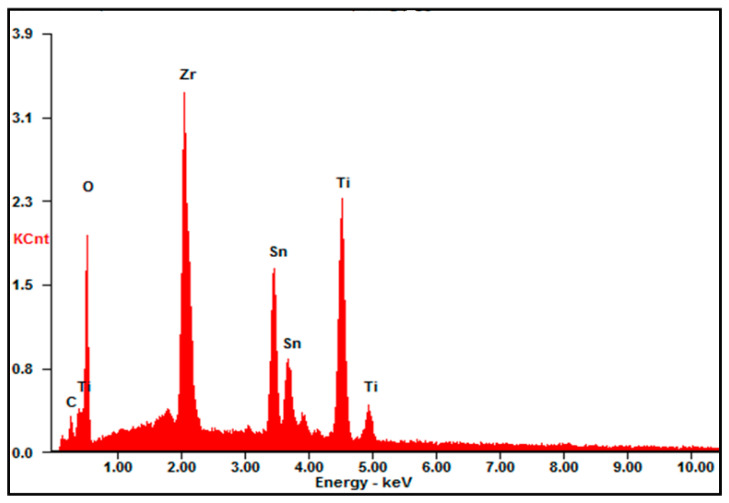
EDX spectrum of ZSTS composite nanofibers.

Figure 2 shows FESEM and TEM micrographs of the pure TiO_2_ and ZSTS composite nanofibers through different magnifications. Figure 2a shows that the surface of the pure TiO_2_ nanofibers is quite smooth and has a diameter of around 200 nm, which is also confirmed by the TEM image (Figure 2b). The low- (Figure 2c) and high- (inset Figure 2c) magnification FESEM micrograph shows that the ZSTS composite is mainly composed of perforated nanofibers with an average diameter of about 150 nm, authenticated by the TEM image as well (Figure 2d). It possesses rough and fibrous surfaces, which are built up of nanoparticles on the surface of the nanofibers. Figure 3 gives a typical EDS spectrum of ZSTS nanofibers, signifying that the nanocomposite comprises Ti, Sn, Zr and O.

**Figure 4:**
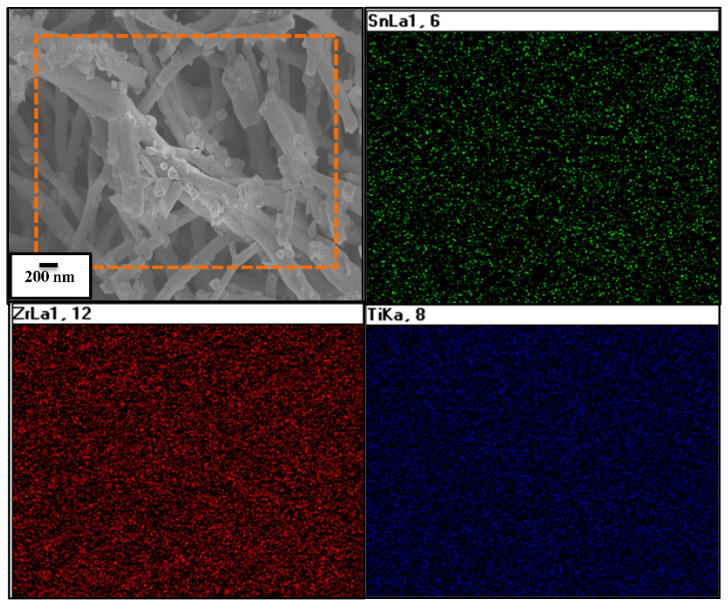
EPMA pattern of ZSTS nanofibers.

Furthermore, the elemental mapping shown in Figure 4 confirms that the electrospun nanofibers have been formed of Zr_0.5_Sn_0.5_TiO_3_ and SnO_2_ materials (Figure 4). The electron probe microanalysis (EPMA) profile shows that the composite possesses Zr, Sn and Ti elements. The elemental mapping picture evidently demonstrates that Zr, Sn and Ti are consistently dispersed all through the surface of the ZSTS composite nanofibers.

**Figure 5:**
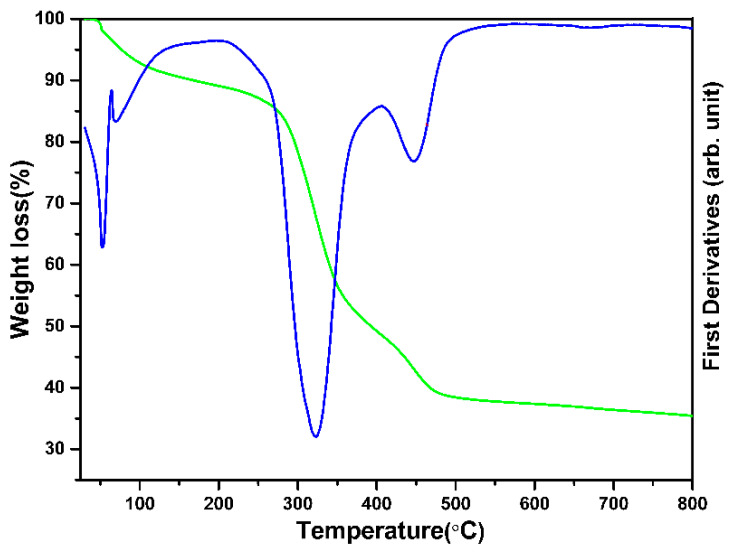
TGA spectra and first derivatives of the as-synthesized ZSTS nanofibers (Green color line shows the weight loss whereas, the blue color shows the first derivatives).

Figure 5 depicts the TGA thermogram of the composite nanofiber precursors from room temperature to 800 °C. The TGA illustrates that the ZSTS composite sample has a total weight reduction of ~64%. The evaporation of water and solvent at temperatures between 25 and 200 °C was the primary cause of the first weight loss (~11%). After 200 °C, the weight loss increased to more than 40% because of the PVAc polymer degrading up to 400 °C, with a significant exothermic peak occurring at 325 °C. The final weight loss (~13%) at temperatures between 400 and 500 °C was caused by the complete disintegration of PVAc, the degradation of the metal salt in the composite and the precipitation of the metal oxide.

**Figure 6:**
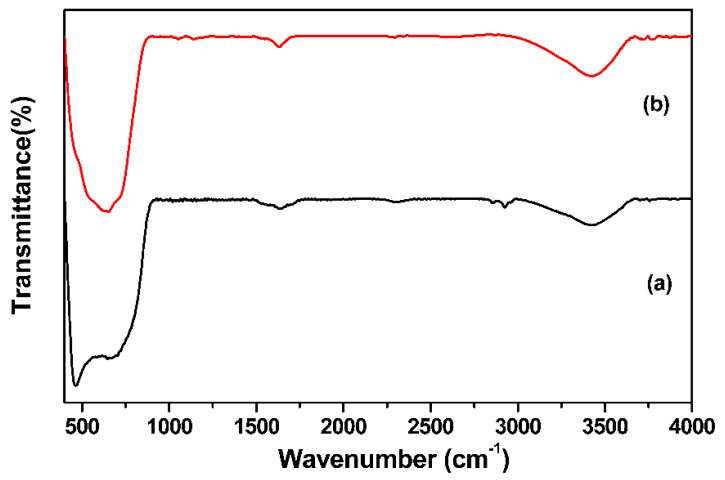
FT−IR spectra of (a) TiO_2_, and (b) ZSTS nanofibers.

In the IR spectra of TiO_2_, a broad band at 3403 cm^−1^, which is associated with the stretching mode of the OH group, and a minor band at 1631 cm^−1^, which denotes the bending mode of molecular water, were visible (Figure 6a). Characteristic peaks at 480 and 616 cm^−1^ indicate the bending and stretching mode of the Ti–O bond [37]. Figure 6b displays the FT–IR spectrum of the heterostructured ZSTS. It shows that the observed wide vibrational band (centered around ~634 cm^−1^) could be defined as a metal–oxygen (M–O) stretching vibration [38].

**Figure 7:**
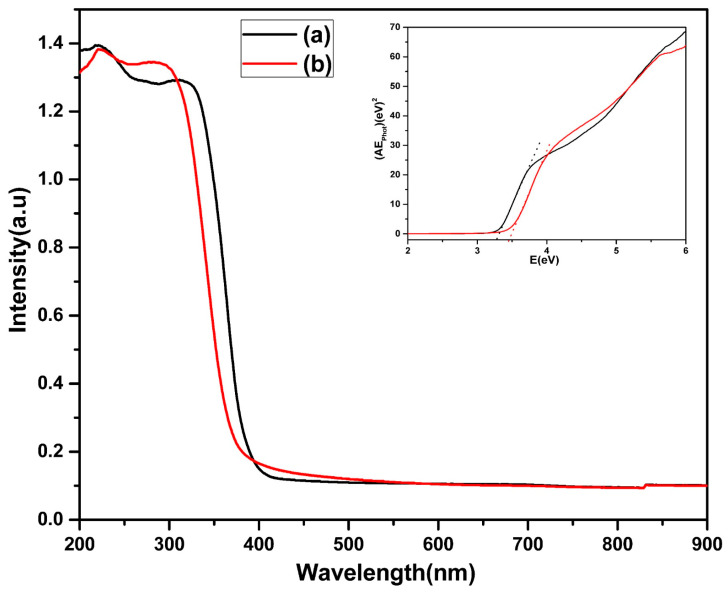
UV–Vis diffuse reflectance spectra of (a) TiO_2_, and (b) ZSTS nanofibers (inset shows plot of Kubelka–Munk function vs. photon energy).

The absorption of light obtained from the diffuse reflectance UV is displayed in Figure 7. The absorption edge for both the samples falls in the UV region (λ < 400 nm). A blue shift in the spectra of the ZSTS nanocomposite is observed compared to the TiO_2_ photocatalyst. The presence of zirconium and tin oxide, which have a wider band gap than titania, may be responsible for the shift in the intensity of the light absorption in the composite sample. To estimate the bandgap energy (E_g_) of the samples, E_g_ was calculated from the plot of (αhν)^1/2^ versus the energy of the stimulating light (hν) using equation αhν = A(hν − Eg)^n/2^ [39]. As a result, the band gaps of the TiO_2_ and ZSTS nanofibers were determined to be 3.28 and 3.45 eV, respectively (inset Figure 7).

**Figure 8:**
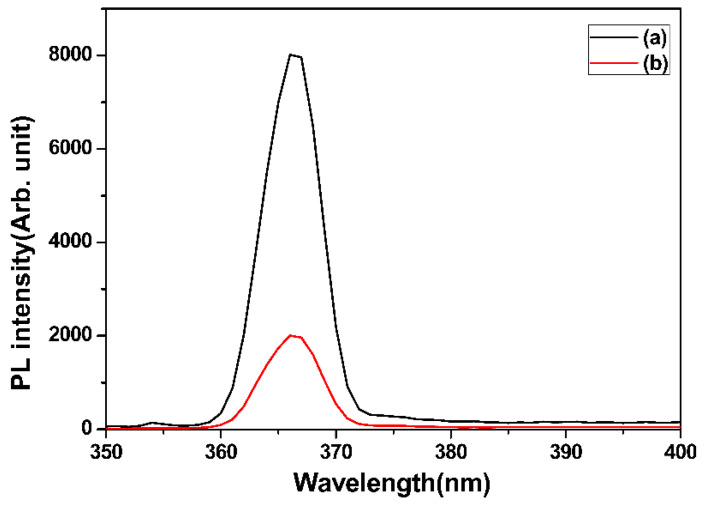
PL spectra of (a) TiO_2_, and (b) Zr_0.5_Sn_0.5_TiO_3_/SnO_2_ nanofibers.

Figure 8 shows the prepared ZSTS nanofibers’ PL spectra. Each sample has an emission peak with a central wavelength of roughly 366 nm. The ZSTS composites exhibit considerable quenching of the PL when compared to TiO_2_. The strength of the PL peaks, which are connected to the recombination of the electron–hole pairs within the semiconductor, further suggests that there is an effective charge transfer within the ZSTS nanofibers. The photogenerated electron–hole pairs can readily transfer at the heterostructured interface in the case of the ZSTS composite, leading to greater photocatalytic activity when exposed to UV light.

**Figure 9:**
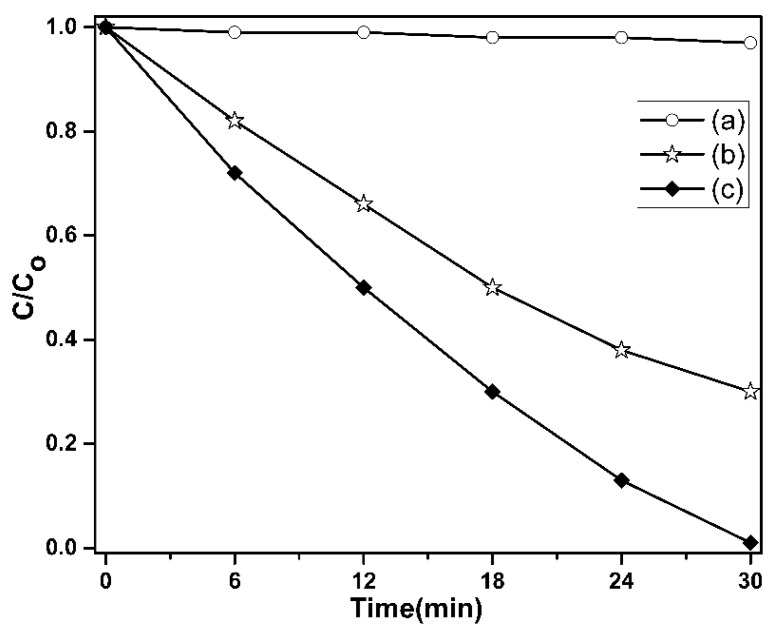
Photocatalytic degradation of RhB dye solution (a) without using TiO_2_ and ZSTS, (b) with TiO_2_, and (c) ZSTS nanofibers.

The photocatalytic activity of the pure TiO_2_ and ZSTS composite was assessed using the photodegradation of RhB dye under UV light irradiation, as shown in Figure 9. For comparison, RhB degradation without the photocatalyst was also carried out. The outcomes showed that RhB degradation was extremely slow in the absence of the photocatalyst. The initial concentration of the RhB dye solution used was 20 ppm. Pure TiO_2_ decolorizes RhB by 70% in 30 min. The ZSTS composite nanofibers, however, show a noticeably improved photocatalytic performance. The composite sample demonstrates that all of the dye can be broken down within 30 min. The experimental findings showed that ZSTS composite photocatalysts have greater photocatalytic activity than pure TiO_2_ photocatalysts. This improvement in photocatalytic efficiency could be due to the heterostructure that forms when Zr_0.5_Sn_0.5_TiO_3_ and SnO_2_ come together, since it is crucial for the separation of electron–hole pairs. Therefore, it seems more likely that the superior photocatalytic activity of ZSTS is attributable to a more effective separation of the electron–hole pairs or to a significantly reduced propensity for electron and hole recombination.

Numerous studies have also documented how combining TiO_2_ with SnO_2_ increases their photocatalytic activity. Owing to the charge separation that takes place at the interface between two semiconductors, this improvement is attributed to a drop in the rate of electron–hole recombination [40]. In addition, it has been discovered that the inclusion of Sn^4+^ ions in the TiO_2_ lattice increases the parent oxide’s exterior acidity [41], which may boost the photocatalytic activity. Also, a superior interaction with surface water tales place in the case of the TiO_2_/ZrO_2_ photocatalyst, which results in the detection of more O_2_H^•^ radicals. However, in the case of TiZr, the heteroatom takes part in the electron trapping sites that result in the establishment of Zr^4+^–O_2_^•–^ species, and this possibly has an impact on the inherent photoactivity of matter [42].

**Figure 10:**
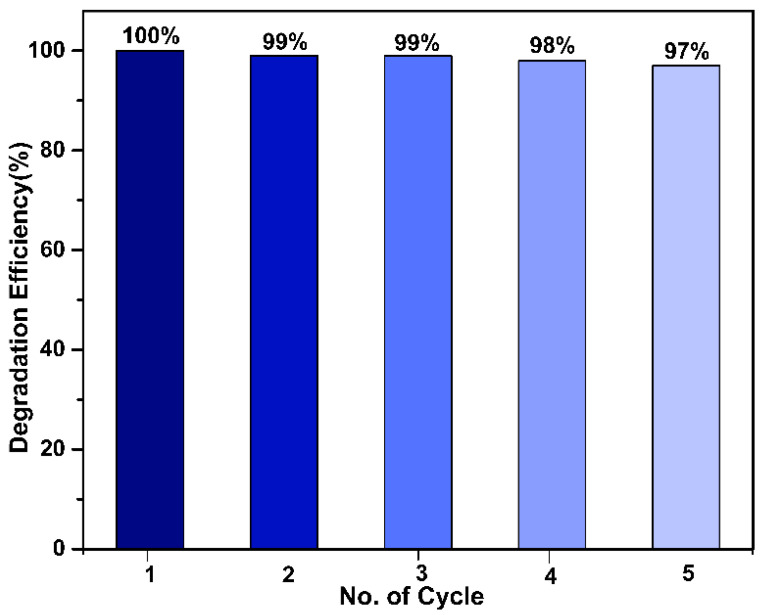
Photocatalytic degradation of RhB dye by ZSTS nanofibers in number of cycles.

To evaluate the reusability of the composites from the standpoint of practical applications, the ZSTS nanofibers were selected. The catalysts currently in use are easily separated from the solution and effectively dispersed when stirred. Centrifugation makes it easy to separate the catalyst from the cleansed solution. The dye did not adhere to the catalyst permanently; rather, photodegradation is what causes the cleaning. The ZSTS composite showed good catalytic stability, keeping a similar level of reactivity even after five cycles (97%) (Figure 10). The unavoidable catalyst loss during recycling should be the cause of the minor reduction in efficiency.

**Figure 11:**
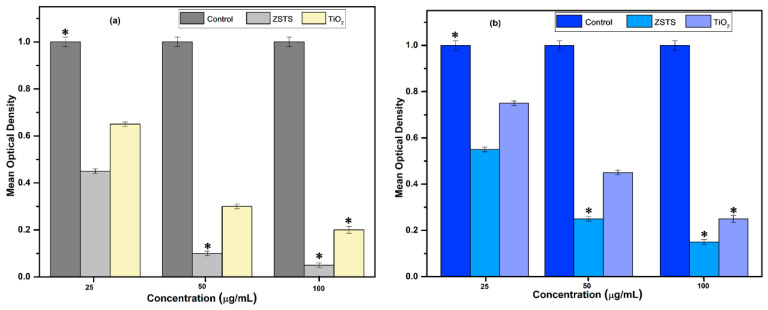
Antimicrobial susceptibility test of TiO_2_ and ZSTS nanofibers (0–100 µg/mL) against (**a**) *E. coli* and (**b**) *S. aureus*. Data represent the mean values ± standard deviation of three replicates. * *p* < 0.005 vs. control.

Antibiotic resistance has incurred staggering health care expenditures in the community over the last decade, necessitating immediate action. This demands a thorough examination of the innovative possibilities. With the ability to autonomously control and manipulate both atoms and molecules, nanotechnology is a multidisciplinary field of study that has enabled significant advancements in numerous scientific fields [43,44]. In the present study, the antibacterial efficacy of TiO_2_ and ZSTS composite nanofibers against two typical bacteria was demonstrated and is shown in Figure 11a. *E. coli*, which frequently causes severe diarrhea, and *S. aureus*, an opportunistic pathogen that frequently causes respiratory infections and food poisoning, are the bacteria that were studied. By treating them using ZSTS nanofibers and the pure TiO_2_, bacterial growth as an indicator of turbidity was observed and computed in order to assess their antibacterial capabilities. The outcomes attained point out that the ability of TiO_2_ nanofibers to inhibit the growth of bacteria is exclusively associated with its selected concentration and UV irradiation, comparable to previous outcomes [33]. In UV/TiO_2_ organization, investigators have proposed diverse elimination mechanisms, including negative effects on deoxyribonucleic acid, membrane impairment that causes the cell contents to leak out, and observed reductions in respiratory operations owing to the loss of precursor enzymes [33]. Nevertheless, there is still a dearth of concrete proof regarding photocatalytic death. In particular, aqueous suspensions produced reactive oxygen species (ROS) when the TiO_2_ solution was exposed to UV radiation. A hole (h^+^) is produced in the valence band via charge separation, while an electron (e^−^) is produced in the conduction band. Hydroxyl radicals (OH) are produced at the surface of the excited particle by elusive electrons in the valence band holes of water and/or hydroxyl ions. The superoxide anion (O^−^_2_) is created when electrons reduce oxygen. The ROS produced by UV-irradiated TiO_2_ nanofibers may have initially interacted with the cell wall, produced free radicals that can enter into the interior of the cell, disrupt the internal contents and cause the cells to deform, leading to disorganization and leakage [33]. In the current investigation, it was found that, in comparison to the pure TiO_2_ nanofibers, the ZSTS nanocomposites demonstrated action against bacterial growth that was more severe (Figure 11b). The current findings suggest that ZSTS nanocomposites may kill certain bacteria and interact with them robustly at a high concentration after 12 h of incubation, regardless of the mechanism involved. However, when the concentration of ZSTS nanocomposites in the suspension was reduced to 25 µg/mL, no palpable antibacterial activity was detected. The shape, increase in surface area [45] and combined outcome are attributed to the amplified activity of the ZSTS nanocomposites. The ZSTS nanofibers in this instance showed significant antibacterial activity against *E. coli*. Additionally, it was discovered that ZSTS nanocomposites and TiO_2_ nanofibers make *E. coli* more sensitive. The two strains’ different cell structures have been shown to be the cause of increased sensitivity [45,46]. The shape of microorganisms is primarily responsible for variances in their sensitivity. Gram-negative bacteria’s cell walls are made up of an exterior lipid membrane and a thin layer of peptidoglycan. ROS, which cause the cells of microorganisms to die, are produced as a result of the effectiveness of the metal oxide. Thus, oxidation and cell death happen due to ROS targeting several distinct locations and biomolecules in the respective microorganisms. However, *S. aureus*, a Gram-positive bacterium, has a dense covering of peptidoglycan that prevents nanoparticles from easily entering *S. aureus*. It could be interpreted that in the case of Gram-negative bacteria, the desired outcome may be achieved via the electrostatic attraction between negatively charged bacteria and positively charged metal ions, which causes bacteria to rupture and die. A similar interpretation of ZrO_2_ nanoparticles has been reported elsewhere [47].

**Figure 12:**
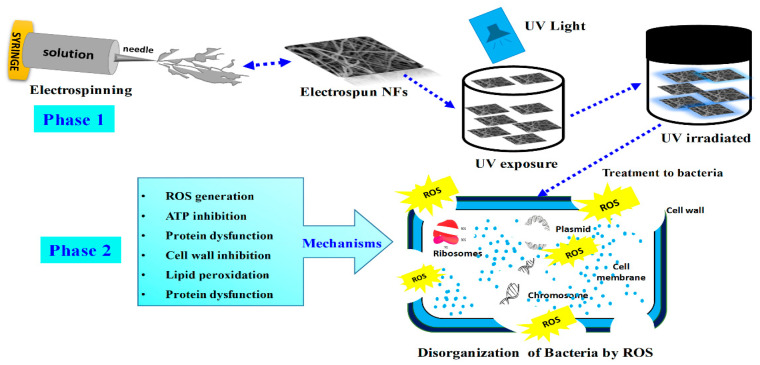
Schematic illustrations of fabrication of ZSTS nanofibers via electrospinning and the bactericidal mechanism thereof.

Previous researchers have documented the bactericidal effects of cotton treated with ZrO_2_ and ZrO_2_ nanoparticles against infections [48]. In yet another investigation, the antibacterial uses of ZrO_2_ NPs in tooth decay assertions were identified [7]. According to previous studies, ZSTS nanocomposites may exaggeratedly increase production of ROS, boost ATPase activity, and cause the expulsion of proteins and electrolytes, leading to the death of bacteria [49]. As such, ZSTS nanocomposites are a unique material that may be utilized for the production of antimicrobial composites. ZSTS nanocomposites have a strong ability to kill bacteria, making them a suitable material for the fabrication of novel bactericides and expanding their range of possible uses. The likely mechanism is shown in Figure 12. Although ZSTS nanocomposites have demonstrated effective antibacterial activity, more research is required to determine their other physicochemical characteristics before they can be employed with confidence. In the end, according to the preliminary information gathered herein, ZSTS nanofibers might be employed topically to regulate the spread of bacterial toxicities and in order to manage the purification of industrial and sewage wastewater.

## 4. Conclusions

In summary, TiO_2_ and ZSTS nanofiber photocatalysts were prepared via the electrospinning method. The experimentation evaluating the photodegradation of RhB and PL investigation revealed that ZSTS nanofibers have a noteworthy effect on the separation of photogenerated charge carriers and UV light photocatalytic action. Furthermore, in 30 min, the composite completely bleached the colorant. The produced photocatalysts also exhibited stability when exposed to UV light. The synergetic effects between Zr_0.5_Sn_0.5_TiO_3_ and SnO_2_ were mostly responsible for the increased photocatalytic activity of the ZSTS composites. The above findings contribute a very effective and robust photocatalytic material for eliminating biological and chemical pollutants, in addition to offering updated knowledge on how to develop some effective ceramics/metal oxide nanofibrous photocatalysts. Interestingly, the ZSTS demonstrated efficient antimicrobial potential for both Gram-negative and Gram-positive bacteria; however, due to their negatively charged cell surface and scavenging activity, these photocatalysts were more vigorous against Gram-negative bacteria. Despite the substantial advancements, there are still a number of problems associated with antibacterial nanofibers that need to be resolved. First, it is necessary to attempt the manufacturing of composite antibacterial nanofibers on a large scale, as it is currently primarily restricted to the laboratory, and to investigate the biocompatibility of these high-aspect-ratio nanofibers with human cell lines. Therefore, the ZSTS could act as new material for environmental applications.

## Data Availability

The data presented in this study are available in article.
